# A commentary on “Efficacy and safety of bicompartmental versus total knee arthroplasty for treating medial tibiofemoral compartment combined with patellofemoral compartment osteoarthritis: a systematic review and META-analysis”

**DOI:** 10.1097/JS9.0000000000003190

**Published:** 2025-08-20

**Authors:** Tianchi Jin, Longhao Chen, Zhen Liu

**Affiliations:** a Zhuji Traditional Chinese Medicine Hospital, Shaoxing, Zhejiang, China; b The Third School of Clinical Medicine (School of Rehabilitation Medicine), Zhejiang Chinese Medical University, Hangzhou, Zhejiang, China

*Dear Editor*,

As we all know, total knee arthroplasty (TKA) significantly improves pain and knee function in patients with osteoarthritis of the knee; however, approximately 10–20% of patients who undergo TKA report dissatisfaction with their postoperative outcomes^[1]^. The procedure replaces all three compartments of the knee and may unnecessarily sacrifice the lateral compartment and cruciate ligament, thereby affecting the knee’s movement patterns, function, and mobility^[2]^. In recent years, bicompartmental knee arthroplasty (BKA) has gained attention for its precision and minimally invasive nature. BKA combines joint function preservation with bicompartmental debridement, allowing replacement of only the damaged compartment while preserving as much of the patient’s knee anatomy as possible. We recently read with great interest the systematic review and meta-analysis published in the I*nternational Journal of Surgery* by Yu Zhang *et al*. This review systematically compared knee function, quality of life, complications, and operative time between BKA and TKA in patients with medial-patellofemoral osteoarthritis (MPFOA). The conclusion suggests that BKA is superior to TKA in terms of postoperative mobility and some functional indices without an increased risk of complications, and provides stage-by-stage evidence for the feasibility and value of the BKA procedure in clinical practice. However, there are a few problems with this paper. Additionally, we employed artificial intelligence to summarize and linguistically enhance these questions^[3]^.

First, although the authors mention in the Methods section that they have completed the PROSPERO registration, the registration number is not explicitly listed in the text, nor is there a link to it, which is not in line with the current requirements for transparency in systematic reviews. We suggest that the authors add the registration number in the text.

Second, differences in BKA procedures were not analyzed. This study failed to differentiate between technical elements, such as the type of prosthesis used in BKA (modular versus integrated prosthesis) and whether robotic-assisted surgery was employed. Modular prostheses are at risk of micromotor interface wear, and integrated prostheses are prone to patellar trajectory abnormalities. These technical differences can affect the confidence of the results. What’s more, BKA is a complex operation with a significant operator learning curve, and operator experience has a direct impact on prosthesis positioning and complication rates. Most of the studies included in this paper were single-center studies that lacked systematic assessment of operator proficiency and technical variability, a key confounding factor that was not adequately considered and may affect the external generalizability of the results.

Third, there was also a high degree of internal heterogeneity in the TKA group. Whether the prosthesis used for TKA is posterior cruciate ligament preserved, posterior cruciate ligament substitute, or posterior cruciate ligament replacement is not specified in the paper^[4]^. Prostheses with different design concepts or from different manufacturers vary significantly in postoperative functional outcomes. Meanwhile, the paper does not specify which osteotomy alignment technique was used by the operator, whether it was mechanical alignment, anatomic alignment, or other alignment techniques^[5]^. In addition, new technologies such as patient-specific instruments, computer-assisted navigation, and robot-assisted surgery, which have emerged in recent years, were not included in detail. These factors may reduce the variability in outcomes between TKA and BKA.

Fourth, this paper did not involve a cost-benefit analysis or a health economics evaluation. The current international field of bone and joint diseases is paying increasing attention to the evaluation of cost-effectiveness ratio, quality-adjusted life year (QALY), and incremental cost-effectiveness ratio (ICER) of surgical procedures, so we suggest that future studies introduce systems economics modeling to assist in policy decision-making^[6]^.

In conclusion, we commend Yu Zhang *et al* for their valuable contribution to knee osteoarthritis, which is of great benefit to patients with patellofemoral osteoarthritis. Our review aims to refine and strengthen the interpretation of their findings. We look forward to the authors’ next study.

## Data Availability

Not applicable.

## References

[1] Zhang R Shen X Yan K Zhang X Zhu C. Comparative efficacy and safety of bicompartmental versus total knee arthroplasty: a systematic review and update meta-analysis. J Orthop Surg Res 2025;20:237. 40045336 10.1186/s13018-024-05384-6 PMC11881321

[2] Blasco JM Hernández-Guillen D Domínguez-Navarro F Acosta-Ballester Y Alakhdar-Mohmara Y Roig-Casasús S. Sensorimotor training prior total knee arthroplasty and effects on functional outcome: a systematic review and meta-analysis. Gait Posture 2021;86:83–93. 33711615 10.1016/j.gaitpost.2021.03.001

[3] Agha RA Mathew G Rashid R. Transparency In The reporting of Artificial Intelligence – the TITAN guideline. Prem J Sci 2025;10:100082.

[4] Lewis PL W-Dahl A Robertsson O. The effect of patient and prosthesis factors on revision rates after total knee replacement using a multi-registry meta-analytic approach. Acta Orthop 2022;93:284–93. 35113168 10.2340/17453674.2022.1997 PMC8808477

[5] Karachalios T Komnos GA. Individualized surgery in primary total knee arthroplasty. EFORT Open Rev 2020;5:663–71. 33204509 10.1302/2058-5241.5.190085 PMC7608523

[6] Goudazi Z Jafari M Kiyaei A Ravangard R Hashemi SA Keshavarz K. Cost-effectiveness analysis of robotic-arm assisted total knee arthroplasty (TKA) versus conventional TKA in Iranian population. Health Econ Rev 2025;15:54. 40613999 10.1186/s13561-025-00648-1 PMC12228254

